# Preface to the Special Issue “Glutathione: Chemistry and Biochemistry”

**DOI:** 10.3390/molecules28165993

**Published:** 2023-08-10

**Authors:** Pál Perjési

**Affiliations:** Institute of Pharmaceutical Chemistry, University of Pécs, H-7624 Pécs, Hungary; pal.perjesi@gytk.pte.hu

This year we celebrate the 135th anniversary of the discovery of glutathione (L-γ-glutamyl-L-cysteinyl-glycine). The major intracellular thiol compound was first described in the literature in 1888 by J. De-Rey Pailhade [1]. He named the substance *phylothion*, the Greek expression for sulfur-loving. Since then, chemists, biochemists, and medical professionals accumulated a wide range of information about this molecule’s cellular and organizational functions. The broad interest in glutathione-related topics is reflected by several recent reviews [2,3,4].

It has been known that endogenous glutathione content and its speciation plays a role, among others, in redox homeostasis, cell cycle control, immunological defense, and pathological abnormalities. Among the latter, hemolytic anemia, cardiovascular diseases, neurological disorders, and distinct types of cancer can be mentioned [5]. Furthermore, it plays a significant role in the biotransformation of drugs and other endogenous or exogenous electrophilic species. In most cases, such transformations protect cellular nucleophilic sites and eliminate the target molecules [6]. Most of these cellular functions are related to the thiol (SH) function of the cysteine moiety.

Due to the redox characteristics of the thiol function, the reduced form of glutathione (GSH) is not only a powerful nucleophile but an antioxidant as well. Because of the high cellular level, the GSSG/2GSH couple is the most abundant redox couple in the cells. Changes in the half-cell reduction potential (E_hc_) of the GSSG/2GSH couple appear to correlate with the biological status of the cells: proliferation E_hc_~−240 mV differentiation; E_hc_~−200 mV; or apoptosis E_hc_~−170 mV [7]. Although it does not mean that the actual redox potential of the GSSG/2GSH system is a determining factor of the cells’ fate [8], the correlations are remarkable and worth further investigation.

Recent clinical trials indicated that oral GSH supplements can elevate body stores of glutathione and markers of immune function [9,10]. The increased demand for pharmacopeial grade glutathione signals the importance of new, economical biotechnology-based technologies for the production and pharmacopeial qualification of glutathione preparations [11]. Additionally, drug delivery systems enhancing the bioavailability of oral glutathione are becoming an important issue. Besides using qualified glutathione (GSH) products as a pharmakon, it can be successfully applied in various other industries where the compound’s reversible redox and antioxidant properties can be utilized.

This book presents the publications that appeared in the Special Issue of Molecules, “*Glutathione: Chemistry and Biochemistry*”. The contributions provide current information on three fields of glutathione research. The first three contributions [12,13,14] review the present-day knowledge of the GSH/GSSG system and the essential GSH-related proteins involved in controlling various cellular events.

The following four contributions [15,16,17,18] present selected interventions which modulate the GSH/GSSG system. One of the contributions of this session [15] describes a new HPLC/DAD method to quantify the reduced glutathione (GSH) and oxidized glutathione (GSSG) levels in rat brain.

The third session involves three [19,20,21] contributions demonstrating the role of GSH in the metabolism of different candidate and clinically used anticancer drugs. One of the contributions [22] a theoretical work, provides valuable information for developing GSH analogs with high ACE inhibitor activity.

By purpose and content, this Special Issue is addressed to the vast number of life science researchers (academic and industrial) and medical professionals who are interested or already engaged in research that involves glutathione.
